# Late-onset sepsis treatment in very preterm infants alters longitudinal microbiome trajectory with lower abundance of *Bifidobacterium* despite probiotic supplementation

**DOI:** 10.1080/19490976.2025.2523808

**Published:** 2025-07-06

**Authors:** David Brian Healy, Shuo Wang, Dhrati Patangia, Ghjuvan Grimaud, R. Paul Ross, Catherine Stanton, Eugene Michael Dempsey

**Affiliations:** a APC Microbiome Ireland, University College Cork, Cork, Ireland; b Department of Paediatrics and Child Health, University College Cork, Cork, Ireland; c Teagasc Food Research Centre, Moorepark, Fermoy, Ireland; d INFANT Research Centre, University College Cork, Cork, Ireland

**Keywords:** Microbiome, prematurity, microbial load, absolute abundance, *Bifidobacterium*, antibiotics

## Abstract

**Introduction:**

Taxonomic instability within the dynamic gut microbiome of very preterm infants can be associated with various adverse outcomes. This longitudinal study was designed to follow the trajectory of microbiome composition and abundance in a cohort of probiotic supplemented very preterm infants with and without sepsis.

**Methods:**

Stool samples (*n* = 180) from probiotic-supplemented participants with culture-positive sepsis (*n* = 8) and matched healthy controls (*n* = 10) were analyzed using 16S rRNA sequencing. Calculation of total copy number per gram (TCN/g) by DNA spiking provided estimates of total microbial load.

**Results:**

TCN/g was significantly different between infants with and without sepsis, the latter having more rapid increase and overall higher TCN/g. In adjusted analysis, sepsis was associated with a significant abundance of *Escherichia-Shigella* (*p* = 0.02) and *Veillonella* (*p* = 0.01). Microbial load and composition appeared to fluctuate following antibiotic administration. Analysis of pre-sepsis samples showed a non-significant trend toward lower *Bifidobacterium* abundance and higher *Escherichia-Shigella* abundance in infants with subsequent sepsis. Antibiotic administration was independently associated with significantly lower (on average 250-fold lower) *Bifidobacterium* (*p* = 0.005) abundance, which remained significant after adjustment for confounders.

**Conclusions:**

Estimation of absolute abundance reveals fluctuations and blooms in key genera within the gut microbiome of very preterm infants that may not be recognized using relative abundance alone. Very preterm neonates with sepsis have a significantly different longitudinal trajectory of microbiome development, which may, in part, extend to lower *Bifidobacterium* and higher *Escherichia-Shigella* abundance prior to the onset of sepsis. *Bifidobacterium* abundance appears to be particularly affected by antibiotic administration compared to other genera.

## Introduction

The gut microbiome of very preterm infants in early life is complex and dynamic. It is thought that, in healthy term newborns, it follows a typical successional development over time resulting in eventual stabilization with *Bifidobacterium* as the dominant genus within the community^1^, particularly in the first 6 months of life. Disruption to the composition of the microbiome can result in temporal lability in the proportionally dominant species. Such instability in the gut microbiome is reported to be associated with complications of prematurity such as necrotizing enterocolitis (NEC) and late-onset sepsis (LOS).^2^ Sepsis events are common in neonatal units, resulting in frequent empiric and therapeutic antibiotic exposure. The incidence of sepsis ranges between 5 and 15% for infants born at less than 32 weeks depending on gestational age.^3^ The gut microbiome in preterm infants is subject to many external influencers including host physiology, and interventions such as supplementation with probiotics, use of various milks, feeding strategies, and antibiotic administration.^1^ In the absence of probiotic supplementation, very preterm infants are more likely to become colonized with members of the *Enterobacteriaceae*,^1,4^ notably species of the *Klebsiella*, *Escherichia* and *Enterobacter* genera, which become dominant in terms of relative abundance. Probiotic supplementation (containing *Bifidobacterium* species) results in dominance of that genus in the gut microbiome.^1^ It has been previously suggested that achieving *Bifidobacterium*-dominance in the gut microbiome is associated with a lower risk of developing LOS^5^ and that temporal stability in the relatively dominant genus is associated with clinical health.^2^

Current culture-independent sequencing techniques allow determination of relative abundances of genera and/or species within each individual sample collected. While longitudinal assessment of relative abundance can provide detailed information of microbiome compositional dynamics, it does not account for changes in overall biomass of the gut microbiome, nor the absolute size of taxonomic communities within it. This is particularly relevant to preterm babies where the microbiome may be in a very early development phase and very sensitive to external and host factors. In this respect, although stability of the dominant genus is important, it is also likely that the magnitude of the microbiome, or fluctuation therein, has implications both compositionally and functionally which in turn undoubtedly affects microbiota-host interactions.^6,7^ We aimed to describe data estimating absolute abundances for longitudinal trends of microbiome development in a cohort of very preterm infants with and without culture positive sepsis. Using techniques for estimation of overall microbiome biomass and absolute taxonomic abundances described in Wang *et al*.,^8^ we highlight important differences from current assumptions.

## Methods

### Site and participants

This was a nested observational study carried out as part of a larger prospective observational study at Cork University Maternity Hospital between 07 April 2021 and 31 December 2022, the protocol for which was previously published.^9^ Infants were eligible for inclusion if they were born at a gestation less than 32 weeks and there was a decision to provide ongoing intensive care. Exclusion criteria were major congenital anomalies, gastrointestinal anomalies, suspicion of inborn errors of metabolism and inability to obtain informed consent. Written informed consent was obtained for all included participants and the study received ethical approval from the Clinical Research Ethics Committee of the Cork Teaching Hospitals (CREC) before commencement (approval letter ECM 4 (t) 10 November 2020 and ECM 3 (mmm) 9 March 2021). Of the first 50 recruited participants (mother-infant dyads/triads) and prior to any sample processing or sequencing, all infants with a diagnosis of culture-positive sepsis were included. Comparative controls, without culture-positive sepsis, were selected from the remaining group and pragmatically matched to the case individuals by both gestational age and birthweight. All infants in this study received a dual-strain probiotic containing *Bifidobacterium bifidum* NCDO 2203 and *Lactobacillus acidophilus* NCDO 1784 (Infloran®). Each 250 mg capsule of probiotic contained not less than 10^9^ colony forming units each of *B. bifidum* and *L. acidophilus*. This was administered at a daily dose of 250 mg/kg. Probiotic administration was commenced once the infant was deemed by the clinical team to be satisfactorily tolerating enteral feeds (i.e. not developing inability to digest enteral feedings associated with increased gastric residuals, abdominal distension and/or emesis) and sufficient volume of milk was being administered in which to dissolve the probiotic granules. Administration was discontinued at 34 weeks corrected gestational age as per current unit policy. Breast milk fortifier (SMA Nutrition Breast Milk Fortifier for preterm low birthweight infants, Nestle Suisse S.A., Vevey, Switzerland) was introduced once milk volumes reached 80 mls/kg/day. Empiric antibiotic combinations at our institution were benzylpenicillin and gentamicin (early-onset sepsis) and flucloxacillin and gentamicin (late-onset sepsis).

### Laboratory

#### DNA extraction

The genomic DNA was extracted from the infants’ samples using the QIAmp Mini stool DNA extraction kit (Qiagen, USA), following the manufacturer’s instructions with slight modifications, as recommended in prior studies.^10^ Stool sample (0.2 g) was added to approximately 50 mg of pre-sterilized (via autoclaving) zirconia beads of various sizes (Biospec, USA). Subsequently, 1 ml of lysis buffer was added, followed by bead beating to homogenize the mixture. Nucleic acid extraction was carried out as per manufacturer’s instruction.

#### Spike-in bacteria and quantification

*Planococcus* sp. APC 3900 (NCBI: txid3035191) and *Pseudoalteromonas* sp. APC 3896 (NCBI: txid3035187), which were isolated from skin of deep-sea fish, as previously described,^8,11^ were used in this study. These two marine bacteria, one from the *Proteobacteria* phylum and the other from *Firmicutes*, are typically absent from mammalian fecal microbiomes under normal physiological conditions. They are readily distinguishable from bacteria commonly found in the human gut using 16S rRNA gene sequencing. The strains were grown in DifcoTM 2216 marine broth (BD DifcoTM, New Jersey, USA) and incubated aerobically with agitation at 30°C for 24 h. *Pseudoalteromonas* sp. APC 3896 is gram-negative bacteria, whereas *Planococcus* sp. APC 3900 is a gram-positive bacterium. These bacteria are phylogenetically distinct and have known genome-derived gene copy numbers. Known concentrations of DNA of these two species were added (“spiked”) to DNA extracted from participants’ samples. The concentration of the sample DNA and spike-in DNA were measured by the Qubit™ 1X dsDNA HA assay kit and Qubit 4 (Invitrogen, USA).

16S rRNA gene copy numbers of the spike-in bacteria *Pseudoalteromonas* sp. APC 3896 and *Planococcus* sp. APC 3900 were determined based on the following formula: *number of copies (molecules) = (amount of DNA ng × 6.022 × 10^23^ molecules/mole)/(length of dsDNA amplicon × 660 g/mole × 1 × 10^9^ ng/g)*. Microbial load, i.e. the total bacterial cell mass within a sample, was defined as the total DNA copy number of all bacteria per gram of that sample. For samples processed using the bead-beating extraction method, the total copy number was standardized by adjusting for the volume of elution buffer and the weight of the fecal sample.

#### Library preparation

The technical procedure for this method is described elsewhere^8^. 20 ul of sample DNA was separated and mixed evenly with 2.5 ul (concentration = 4.31 ng/μl) *Pseudoalteromonas* sp. APC 3896 and 2.5 ul (concentration = 9.59 ng/μl) of *Planococcus* sp. APC 3900. Qubit 4 was used to quantify the concentration and then samples were normalized to 5 ng/ul with 10 mM Tris before commencing library preparation. In this study, 2.32 × 10^6 copies of *Pseudoalteromonas* sp. APC 3896 and 5.99 × 10^6 copies of *Planococcus* sp. APC 3900 were added to achieve a final relative abundance of the spike-in between 0.1% and 10% in most samples. The DNA samples were subjected to amplification, targeting the V3 and V4 region of the 16S rRNA gene using primers 341F/806 R. This process produced an amplification fragment of approximately 465 bp. Following the manufacturer’s instructions, the amplicons were then prepared for sequencing and analyzed using the Illumina HiSeq 2000 platform with 2 × 300 bp chemistry (Illumina technologies, USA).

### Statistics analysis

The raw reads (paired-end reads) were analyzed using the dada2 pipeline. Denoising and pre-processing of the sequence reads were carried out using the dada2 pipeline,^12^ while filtering and trimming of sequence data was carried out using the core sample inference algorithm. Taxonomy to the Amplicon Sequence Variants (ASVs) was assigned using the SILVA database release 138.1.^13^ Where necessary, data were formally tested for normality.

Diversity analysis was carried out in R using phyloseq and microbiome packages. Regression plots of Shannon diversity, relative abundance and total copy number data were modeled using LOESS (locally weighted estimated scatterplot smoothing) regression and plotted with 95% confidence intervals (CIs) and visualized using ggplot2 package v.3.3.2. Wilcoxon signed-rank test was used for statistical comparison.

To assess for differences in the pre-sepsis microbiome, samples up to five days prior to sepsis were included. For comparison, samples from participants in the Non-Sepsis group were selected that approximated the day of life the sample was collected and the cumulative total of antibiotics received up to that point.

For generation of longitudinal regression curves, the sampling time period was limited to 11 weeks to minimize effect of outlying samples at later timepoints from individual participants. Where weeks were represented by multiple samples, the single latest sample was selected and the others omitted. All samples were used to generate Sankey plots for each individual participant and for linear regression analysis. The “lme4” and “lmerTest” packages were used to perform linear regression for adjustment of timing of sample collection, the cumulative number of days that probiotics had been received prior to sample collection, the cumulative number of days antibiotics had been received, the number of days since last antibiotics received, and birthweight. Participant was used as a random effect in the model to account for repeated measures and log transformation was used to account for zero values.

## Results

In this study, we aimed to follow the trajectory of the microbiome in probiotic-supplemented very preterm babies in healthy versus LOS conditions.

### Participants and samples

To achieve this, 180 samples from 18 infants were included (Sepsis group (*n* = 8), Non-Sepsis group (*n* = 10)). The median (interquartile range (IQR)) number of samples collected was 10 (8 to 12). The median (IQR) number of days to collection of the first sample was 6 (2 to 7) days. The median (IQR) number of days to commencement of probiotics was 5 (5 to 7) days. Breastmilk, specifically mother’s own milk, was the primary milk used. Percentages of mother’s own milk, donor breast milk and formula received were Week 1: 79.5, 15.2, 1.1; Week 2: 92.4, 7.6, 0; Week 3: 83.8, 10.1, 6.1, respectively. Across the whole cohort (*n* = 18), mean (SD) gestational age was lower in the Sepsis group (26.5 (2.8) weeks vs 27.8 (0.7) weeks), and mean (SD) birthweight was 0.92 (0.35) kg and 1 (0.25) kg in the Sepsis and Non-Sepsis groups, respectively. The participant demographics can be found in Supplementary Table S1.

All cases of culture-positive sepsis were diagnosed after 72 h of age and therefore considered LOS . Six of eight cases were coagulase-negative *Staphylococci*. There was one case of *Streptococcus agalactiae* and one of *Staphylococcus aureus*. Two infants also went on to later develop a second sepsis event; one with *Escherichia coli* and one with an extended spectrum beta-lactamase resistant *E.coli*. Antibiotic exposures were varied and depended on type and severity of initial clinical presentation. Details of sepsis and antibiotic exposure can be seen in Table 1.

**Table 1.** Antibiotic exposure and cultured bacteria in sepsis events for both non-sepsis and sepsis groups.

	Antibiotics received during neonatal course (days)												
Participant	B	G	F	V	C	A	M	Met					
**Non-Sepsis Group**													
1	2	7	10	–	0	0	–	–					
2	1	4	2	–	0	2	–	–					
3	2	4	5	–	0	0	–	–					
4	2	3	3	–	3	0	–	–					
5	2	1	0	–	0	0	–	–					
6	2	4	3	–	0	0	–	–					
7	3	2	0	–	0	0	–	–					
8	2	10	11	–	0	0	–	–					
9	3	6	5	–	0	0	–	–					
10	4	7	3	–	0	0	–	–					
	Antibiotics received prior to sepsis event (days)								Sepsis event				
**Participant**	**B**	**G**	**F**	**V**	**C**	**A**	**M**	**Met**	Cultured bacteria	Primary treatment antibiotic(s)	Duration (days)	Empiric/other antibiotics used during sepsis event	Duration (days)
**Sepsis Group**													
11	1	2	0	0	0	2	0	0	*S. epidermidis*	V	11	F,G,C	2
12	4	13	6	0	6	7	0	0	*S. agalactiae*	B,G	11	V,C,A,Met	3
13	4	7	8	0	0	0	0	0	*S. capitis*	C,A,G	20	F,G,V	8
14	2	4	3	1	0	0	9	0	*S. epidermidis*	M	10	n/a	–
15	2	5	3	0	0	0	0	0	*S. epidermidis*	V	10	F,G,C	5
16	0	0	0	0	0	0	0	0	*S. aureus*	F	19	G,V	5
17	0	0	0	0	0	0	0	0	*S. warnerii*	V	13	F,G	2
18	6	4	0	0	0	0	0	0	*S. capitis*	V	10	F,G,A	7

B, Benzylpenicillin; G, Gentamicin; F, Flucloxacillin; V, Vancomycin; C, Cefotaxime; A, Amoxicillin; M, Meropenem; Met, Metronidazole.

For those included in analysis of pre-sepsis samples (*n* = 16), mean (SD) gestational age was 26.5 (2.9) weeks and 27.6 (0.7) weeks and mean (SD) birthweight was 0.84 (0.21) kg and 0.93 (0.22) kg in the Sepsis and Non-Sepsis groups, respectively. In the Sepsis group, positive bacterial culture occurred at a median (IQR) of 12 (9 to 18) postnatal days. The analyzed pre-sepsis samples were collected at comparable days of life in each group (median (IQR) (11 (6 to 18) vs (11.5 (7 to 17)).

### Diversity indices

**Figure 1.**
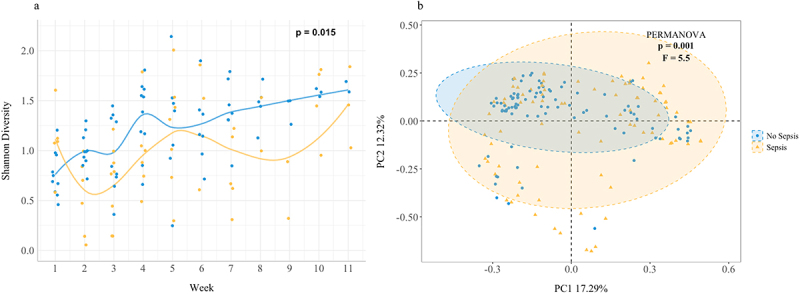
a) Regression plots of Shannon diversity over time for infants with and without sepsis, b) Principle coordinate analysis plot illustrating Bray-Curtis dissimilarities between the sepsis and Non-sepsis groups.

Alpha-diversity (Shannon) increased from birth to the 11th week of life (Figure 1(a)) and was significantly different between the Sepsis and Non-Sepsis groups (*p* = 0.015). While alpha-diversity within the non-sepsis group increased steadily over the sampling period, within the sepsis group it initially decreased. Principle coordinate analysis indicated distinct clustering of samples based on group (*p* = 0.001) despite the differences in timing of the samples (Figure 1(b)).

### Relative abundance

**Figure 2.**
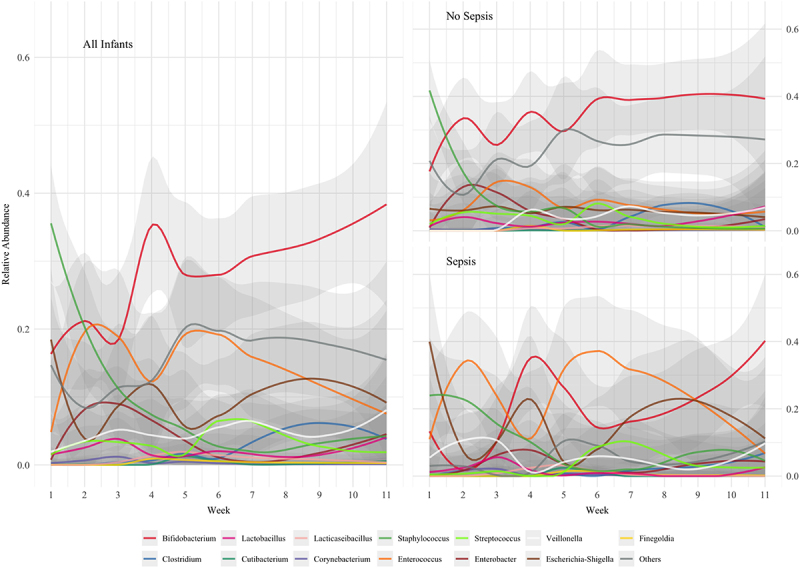
Regression plots of relative abundance of bacterial genera over time for all infants, infants without sepsis, and infants with sepsis.

LOESS regression demonstrated that *Staphylococcus* was predominant in terms of relative abundance in samples collected within the first 10 days of life across the whole cohort (Figure 2). *Enterococcus* relative abundance peaked at the end of the second week and *Bifidobacterium* gradually became the dominant genus after the third week of life with occasional increases in relative abundance of other genera between week 4 and week 10 of life (Figure 2). When grouped by sepsis status, infants in the non-sepsis group showed early dominance of *Staphylococcus* with a decline in relative abundance to low levels in the first two weeks. In these infants, *Bifidobacterium* relative abundance was moderately high from the outset, did not decline, and gradually increased to the sixth week of life and thereafter remained stable. Conversely, in the sepsis group, *Escherichia-Shigella* predominated at the outset and the microbiome was more dynamic with various genera occupying dominant positions in the microbiome over the course of sampling, including *Enterococcus*. In these infants, *Bifidobacterium* did not emerge as a dominant genus until after the ninth week of life.

### Estimation of total abundance

**Figure 3.**
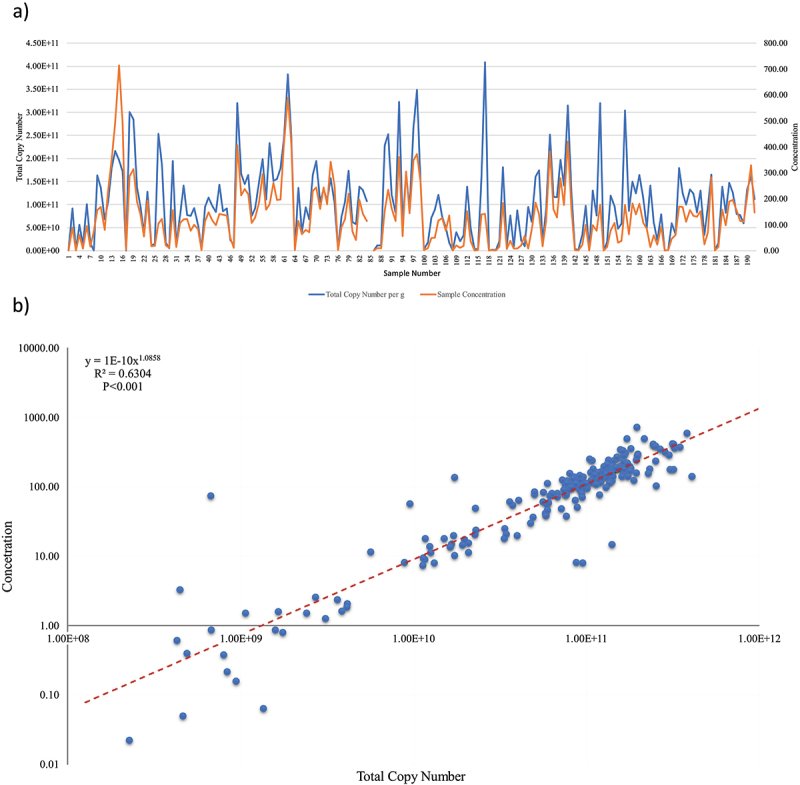
(a) Line graph of sample concentration and total copy number per gram for each individual sample. b) Scatter plot of concentration versus total copy number per gram with correlation trendline.

Sample concentrations ranged from 0.02 to 714.28 ng/µl. Total copy number, after removal of the spiked DNA, ranged from 2.26e8 to 4.08e11 reads/gram. Sample concentration was significantly correlated with total copy number per gram (TCN/g) (R^2^ = 0.6304, *p* < 0.001) (Figure 3). TCN/g was comparable to standardized DNA concentration as an estimation of microbial load in the observed longitudinal pattern of microbiome establishment.

### Absolute abundance, sepsis and antibiotic treatment

**Figure 4.**
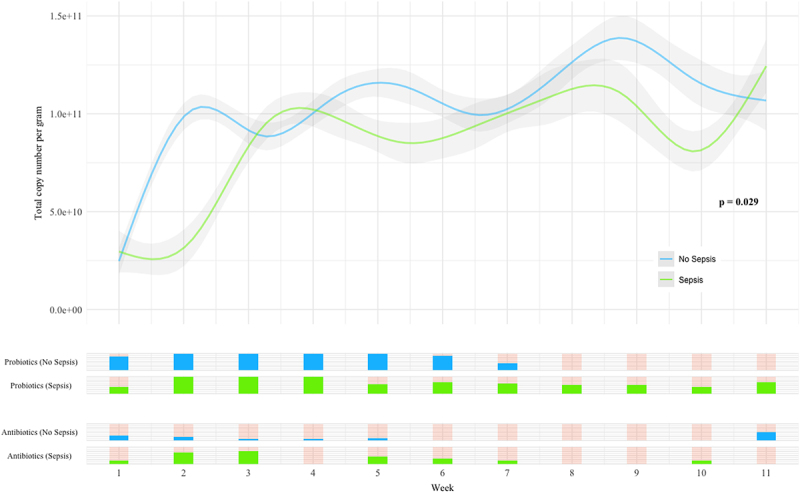
Regression plot showing longitudinal trends in total copy number per gram for infants with and without sepsis. Bands below indicate numbers of infants receiving probiotics and antibiotics over time (broader band indicates more infants).

**Figure 5.**
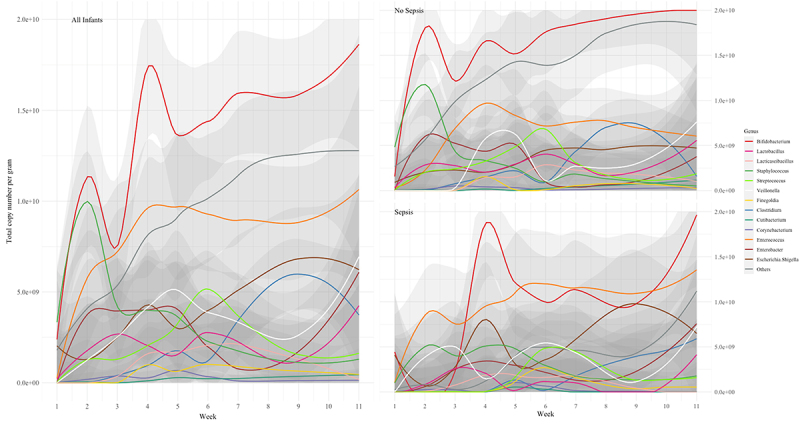
Regression plots of total copy number per gram of bacterial genera over time for all infants, infants without sepsis, and infants with sepsis.

**Figure 6.**
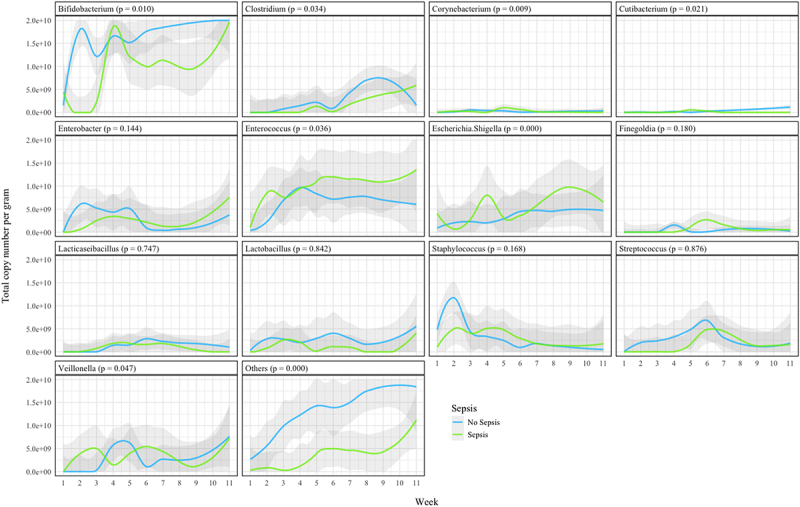
Regression plots of total copy number per gram of bacterial genera. Stars denote significance.

TCN/g increased more rapidly in the Non-Sepsis group compared with the Sepsis group (Figure 4) and the longitudinal trends were significantly different between groups (*p* = 0.029), with earlier increases in microbial load seen in the Non-Sepsis group. Evaluation of TCN/g suggested comparatively higher *Escherichia-Shigella* abundances within the first few days of life in the Sepsis group, replaced by *Enterococcus* as the dominant genus through the second and third weeks. In all infants, and consistent with previous work,^14,15^ *Staphylococcus* was seen to decrease in relative abundance during the first two weeks after birth (Figure 2). However, TCN/g demonstrates that the abundance of *Staphylococcus*, in all infants, and *Bifidobacterium*, in infants without sepsis, started at low levels and rapidly increased in the first week (Figure 5). In infants without sepsis, *Bifidobacterium* became more prevalent than other genera in the microbiome early in the second week, and remained the most abundant thereafter (Figure 5). Individual bacterial genera were significantly different between groups (Figure 6), particularly *Bifidobacterium*, *Escherichia-Shigella*, and the group comprising “Others”.

**Figure 7.**
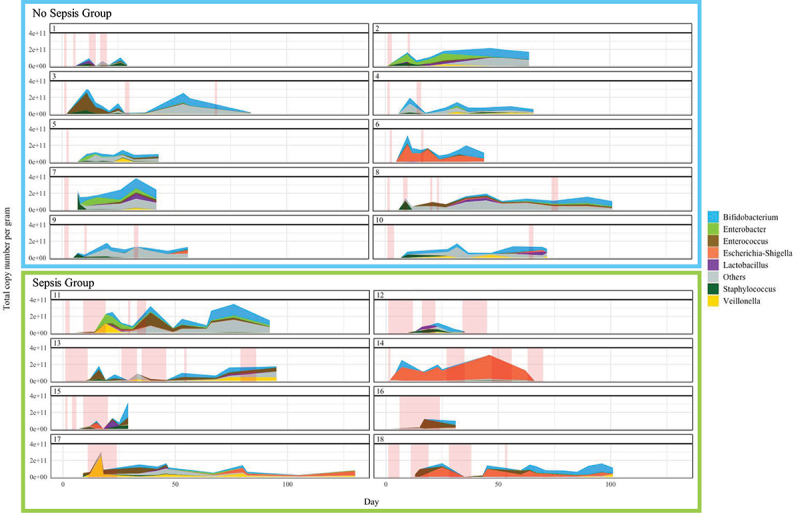
Stacked area Sankey plots illustrating the progression over time of total copy number and the proportion of each component genus in the gut microbiome of each infant.

Evaluation of longitudinal progression of gut microbiome for each individual participant revealed high intra and inter-individual variability both in terms of microbial load variability and gut microbiome composition (Figure 5). Shifts to very low abundance states (i.e. up to 1000-fold reduction in total copy number) were almost always associated with treatment courses of antibiotics, particularly longer courses. Notably, the total abundance of *Bifidobacterium* did not increase when under the selective pressure of antibiotic treatment, whereas changes in abundance of genera such as *Enterococcus*, *Enterobacter*, *Veillonella*, and *Escherichia-Shigella* were inconsistent, with both increases and decreases in total abundance observed during antibiotic treatment (Figure 7).

In unadjusted analysis, there was a significantly lower TCN/g of *Bifidobacterium* in infants with sepsis (OR (95% CI) 0.15 (0.03 to 0.63), *p* = 0.02). This was no longer significant after adjustment (OR (95% CI) 0.38 (0.08 to 1.69), *p* = 0.24). (Table 2). *Staphylococcus*, *Veillonella* and *Escherichia-Shigella* abundances were not significantly different in unadjusted analysis. However, after adjustment these became significant, with *Staphylococcus* abundance being lower in infants with sepsis (OR (95% CI) 0.09 (0.01 to 0.68), *p* = 0.03), and *Veillonella* (OR (95% CI) 27596.65 (51.37 to 1.48e + 07), *p* = 0.01) and *Escherichia-Shigella* (OR (95% CI) 23889.67 (19.28 to 2.98e + 07), *p* = 0.02) demonstrating increased abundance in that group. Other main genera did not differ between the two groups.

**Table 2.** Descriptive, unadjusted and adjusted analyses for differences in total copy number per gram of the main genera in samples collected when on compared with when off antibiotics.

	Off Antibiotics		On Antibiotics		Unadjusted analysis*			Adjusted analysis*^$^		
	Median (total copy number per gram)	IQR	Median (total copy number per gram)	IQR	OR	95% CI	P-value	OR	95% CI	P-value
*Bifidobacterium*	3.43E + 10	4.72E + 10	2.39E + 08	3.47E + 09	0.03	0.01 - 0.11	**0.0000**	0.18	0.05 - 0.58	**0.0053**
*Lactobacillus*	3.64E + 07	5.06E + 08	0.00E + 00	1.19E + 07	0.02	0.00 - 0.55	**0.02**	0.04	0.00 - 1.75	0.10
*Escherichia*-*Shigella*	4.06E + 08	8.85E + 09	6.11E + 07	2.66E + 08	0.04	0.00 - 0.52	**0.01**	0.46	0.04 - 5.73	0.55
*Enterobacter*	0.00E + 00	2.39E + 09	0.00E + 00	0.00E + 00	0.00	0.00 - 0.05	**0.0001**	0.14	0.01 - 2.46	0.18
*Enterococcus*	4.28E + 09	1.62E + 10	2.88E + 08	8.14E + 09	0.08	0.01 - 0.97	**0.048**	2.36	0.20 - 30.07	0.51
*Veillonella*	1.54E + 07	1.63E + 09	6.60E + 06	6.17E + 07	0.25	0.01 - 4.51	0.35	23.80	1.64 - 368.07	**0.02**
*Staphylococcus*	1.04E + 09	4.57E + 09	2.85E + 08	2.51E + 09	0.16	0.04 - 0.64	**0.01**	0.09	0.02 - 0.42	**0.0021**
Others	8.11E + 09	4.52E + 10	4.75E + 08	7.61E + 09	0.08	0.01 - 0.48	**0.01**	0.72	0.11 - 4.88	0.74

*.data log transformed.

$ adjusted for day of life sample collected, number of days since last antibiotic and total days of antibiotic to that timepoint, total days of probiotic to that timepoint, and birthweight.

IQR, interquartile range; OR, odds ratio; CI, confidence interval.

**Figure 8.**
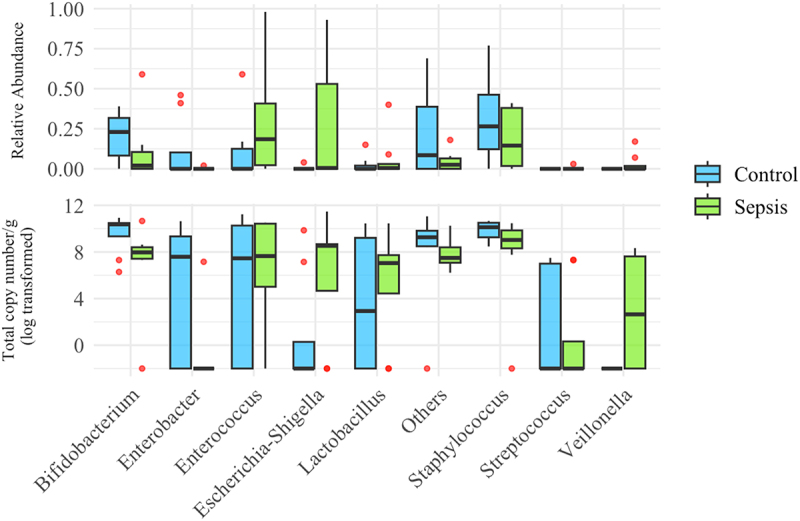
Boxplots of abundance of genera for pre-sepsis samples only.

In analysis of pre-sepsis samples only (Figure 8), the differences between non-sepsis and sepsis groups were not statistically different for any genus. However, it is notable that the median TCN/g for *Bifidobacterium* was 250 times lower in the Sepsis group than in the Non-Sepsis group (9.25e7 vs 2.34e10) and, conversely, the median TCN/g for *Escherichia-Shigella* in the Non-Sepsis samples was 0, while in the samples taken from infants with sepsis, it was 3.4e8.

**Figure 9.**
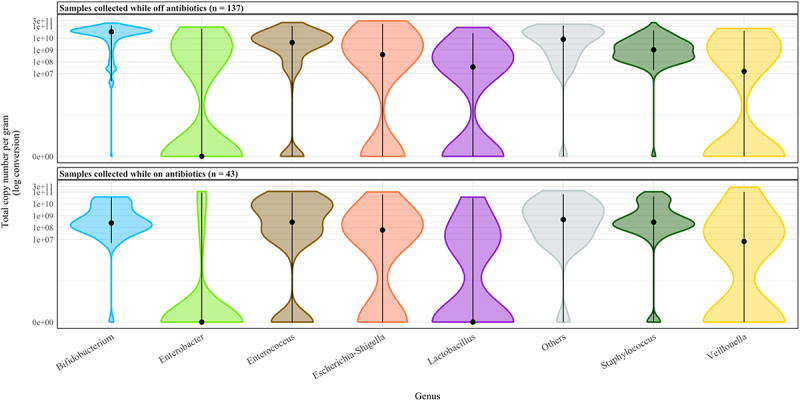
Distribution of total copy number of each genus for all samples collected on and off antibiotics. Median total copy number is demarcated by the black dot.

Unadjusted analysis demonstrated significant differences between *Bifidobacterium*, *Lactobacillus*, *Escherichia-Shigella*, *Enterococcus*, *Enterobacter*, *Staphylococcus* and the combined ‘Others’ genus for samples collected when participants were on antibiotics compared with off antibiotics (Figure 9 and Table 3). After adjustment, antibiotic use was independently associated with a significant reduction in the total copy number of *Bifidobacterium* (OR (95% CI) 0.18 (0.05 to 0.58), *p* = 0.0053) and *Staphylococcus* (OR (95% CI) 0.09 (0.02 to 0.42), *p* = 0.002). There was a statistically significant increase in the TCN/g of *Veillonella* with antibiotic administration (OR (95% CI) 23.8 (1.64 to 368.07), *p* = 0.02).

**Table 3.** Descriptive, unadjusted and adjusted analyses for differences in total copy number per gram of the main genera in samples collected from infants with and without sepsis.

	No Sepsis		Sepsis		Unadjusted analysis*			Adjusted analysis*^$^		
	Median (total copy number per gram)	IQR	Median (total copy number per gram)	IQR	OR	95% CI	P-value	OR	95% CI	P-value
Bifidobacterium	3.20E + 10	4.17E + 10	7.47E + 09	3.92E + 10	0.15	0.03 - 0.63	**0.02**	0.38	0.08 - 1.69	0.24
Lactobacillus	8.47E + 06	3.72E + 08	1.51E + 07	2.00E + 08	3.69	0.21 - 63.36	0.37	6.36	0.09 - 463.50	0.41
Escherichia-Shigella	1.52E + 07	1.34E + 09	5.72E + 08	1.34E + 10	1166.98	1.42 - 9.33E + 05	0.06	23889.67	19.28 - 2.96E + 07	**0.02**
Enterobacter	0.00E + 00	1.53E + 09	0.00E + 00	7.53E + 08	0.02	0.00 - 18.02	0.28	0.14	0.00 - 160.00	0.61
Enterococcus	2.46E + 09	1.28E + 10	5.93E + 09	2.27E + 10	1.75	0.01 - 326.66	0.84	33.27	0.25 - 4331.48	0.19
Veillonella	0.00E + 00	3.03E + 08	6.17E + 07	2.32E + 09	287.72	0.47 - 1.73E + 05	0.10	27596.65	51.37 - 1.48E + 07	**0.01**
Staphylococcus	1.36E + 09	5.61E + 09	3.58E + 08	3.60E + 09	0.34	0.08 - 1.51	0.18	0.09	0.01 - 0.68	**0.03**
Others	1.79E + 10	4.90E + 10	1.81E + 09	9.89E + 09	0.34	0.02 - 5.08	0.45	0.49	0.02 - 12.57	0.68

*data log transformed.

$adjusted for day of life sample collected, number of days since last antibiotic and total days of antibiotic to that timepoint, total days of probiotic to that timepoint, and birthweight.

IQR, interquartile range; OR, odds ratio; CI, confidence interval.

Although supplemented in the same quantity in the probiotic, *Lactobacillus* TCN/g was more likely to be reduced by antibiotic use. *Bifidobacterium* was present in all but two samples collected (both in Participant 14). *Lactobacillus* was frequently absent from samples. It was absent in 37% of samples collected while off antibiotics and 53% of samples collected when on antibiotics.

## Discussion

This observational study is the first to report genus-level longitudinal changes in the bacterial gut microbiome over the first weeks of life of very preterm infants using both absolute and compositional microbiome abundance metrics. In this cohort of infants, with and without sepsis, we have demonstrated significant reductions in the abundances of bacterial genera during antibiotic administration, particularly *Bifidobacterium*. We also described high intra-individual variability in overall total abundance, which often coincided with antibiotic administration.

In our overall cohort, successional relative abundance patterns within the first weeks of microbiome establishment are similar to other published work,^1,16,17^ and our observations are in keeping with the previously described high inter- and intra-individual compositional variability of the preterm gut microbiome. In contrast to full term healthy breastfed infants, the preterm infants in this study had higher relative abundances of Enterobacteriaceae and of Gammaproteobacteria including *Escherichia* and *Enterobacter*. Characteristically, term breastfed infants progress quickly to having bacterial gut microbiomes dominated by *Bifidobacterium*.^14^ With probiotic supplementation, infants in our Non-Sepsis group transitioned quickly to *Bifidobacterium* dominance (Figures 2 and 5). In contrast, infants in the Sepsis group did not progress to *Bifidobacterium* dominance until at earliest the 4^th^ week (Figures 2 and 5). Comparatively, other work on preterm infants not receiving probiotics has demonstrated a high preponderance for Enterobacteriaceae dominance with low relative abundances of *Bifidobacterium*.^4^

Previous work has demonstrated that increased dynamicity in relative abundance of the preterm gut microbiome may be associated with adverse outcome.^2^ We note in the present study, that changes in relative dominance often occurred after a marked reduction in overall total abundance (Figure 7, e.g. Participants 1, 2, 4, 13, 15, 17). This frequently occurred with administration of antibiotics in this cohort. As such, we speculate that the previously described association between adverse outcomes and instability in microbiome dominance structure, that can be associated with adverse outcome such as NEC, may exist because these shifts in dominance, in part, reflect fluctuations in microbiome biomass.

**Figure 10.**
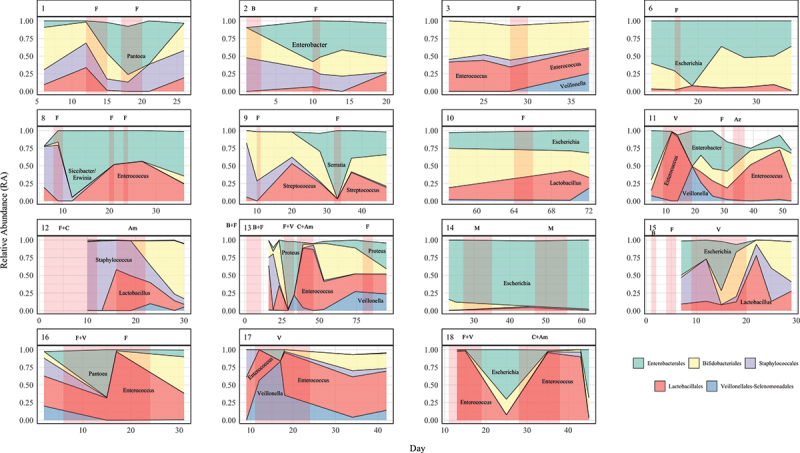
Antibiotic-focussed Sankey plots of the top five most abundant bacterial orders for each participant with sufficient pre and post antibiotic samples. Red opacified areas represent duration of antibiotic administration. Antibiotics administered are denoted above the plot. B, benzylpenicillin; F, flucloxacillin; V, vancomycin; C, cefotaxime; Am, amoxicillin; M, meropenem; Az, azithromycin. Gentamicin was concurrently administered for some duration of each course and as such has been omitted.

**Figure 11.**
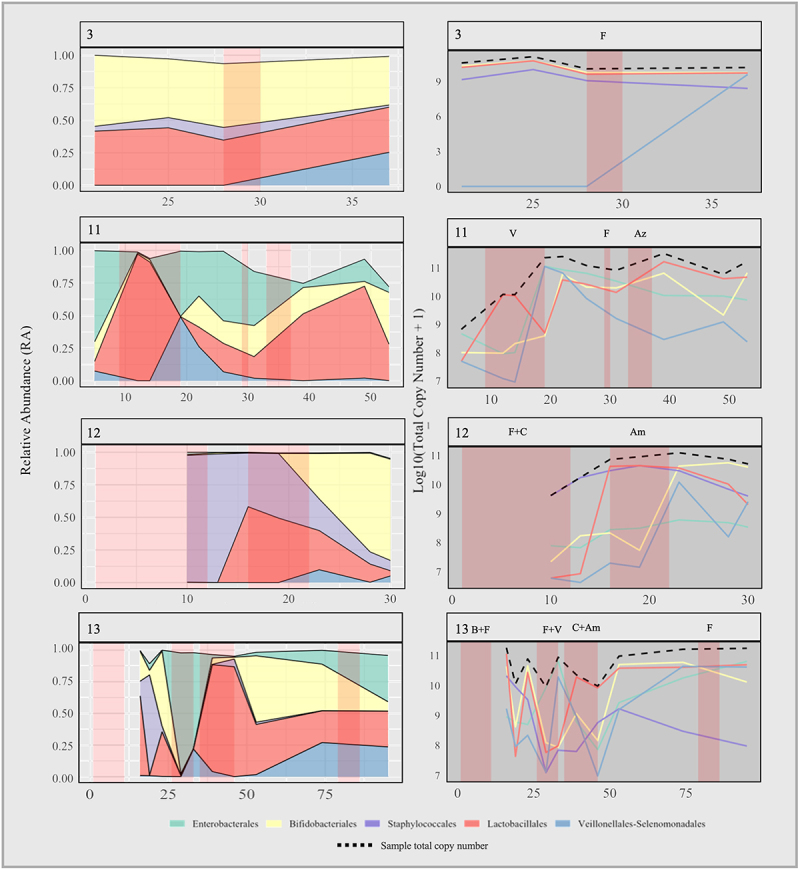
Comparison plot of relative abundance vs log10(TCN/g+ 1) for 4 selected participants. Red opacified areas represent duration of antibiotic administration. Antibiotics administered are denoted above the plot. B, benzylpenicillin; F, flucloxacillin; V, vancomycin; C, cefotaxime; Am, amoxicillin; M, meropenem; Az, azithromycin. Gentamicin was concurrently administered for some duration of each course and as such has been omitted.

Antibiotics were a major influence on gut microbiome dominance patterns in our studied infants. Observed patterns of transition in dominance appeared to follow intrinsic patterns of antimicrobial resistance (Figure 10), although the scope of this study did not allow for formal evaluation of this at the level of antimicrobial resistance genes. We observed that bacteria of the Enterobacterales and Lactobacillales orders were more likely to bloom during or after antibiotics, particularly with administration of more broad spectrum antibiotics such as cefotaxime, vancomycin and amoxicillin. Indeed, in three participants (2, 8, and 14) who received extended courses of vancomycin, a particular pattern of initial dominance of genera of the order Enterobacterales and *Veillonella*, followed by dominance of *Enterococcus*, was noted. The potential for *Enterococcus* sp. to bloom in the gut microbiome after antibiotic-induced perturbation has been previously suggested.^4^ This was also seen in our studied infants, where increases in the relative abundance of *Enterococcus* was often as a result of opportunistic increases in its abundance simultaneous to concurrent decreases in other genera. The resulting large shifts in relative abundance reflected differential changes in TCN/g between genera (Figure 11). *Bifidobacterium* relative abundance was always decreased by antibiotic administration, even where there was uninterrupted concurrent probiotic administration. This did not always reflect a reduction in TCN/g but instead differential changes in abundance between it and other genera. Even though *L. acidophilus* was present in the supplemental combined-probiotic in the same quantity as *B. bifidum*, it was often absent in the samples collected, particularly during intercurrent antibiotic administration. This finding may relate to the strain’s sensitivity to benzylpenicillin, however as intrinsic resistance against gentamicin, flucloxacillin, and vancomycin typically exist in *L. acidophilus* it is possible that the low abundance of *Lactobacillus* in these study participants relates instead to inter-species competition or host incompatibility. While inferences cannot be drawn from this small study, it prompts the need for further research to develop clinical microbiome-rescue strategies around antibiotic administration, such as ensuring the use of mother’s own milk or donor breast milk during this period, or reinstating probiotic administration as soon as possible. These strategies may be important to induce stability in the microbiome, prevent excessive blooms in potentially harmful bacteria,^18^ and to reduce accumulation of bacteria harboring antibiotic resistance genes within the local NICU environment.

The clinical heterogeneity of the very preterm population makes the study of the gut microbiome challenging.^19^ We acknowledge that this study is limited by its small size and by high antibiotic usage in those infants with confirmed sepsis, thus making it difficult to infer differences between infants with and without sepsis with regard to microbiome structure or progression. Nevertheless, important information has been revealed through use of techniques to estimate total bacterial load and estimates of absolute genus abundances. In terms of relative abundance, the preterm gut microbiome is known to have high inter-individual variability^20^ and high temporal variability.^2,5^ The early, establishing microbiome of preterm neonates progresses from a low biomass to high biomass community quickly. However, due to variability in practices such as feeding, probiotic supplementation and antibiotic use, this progress may be impacted in terms of community species structure and their relative proportions. It is likely that these practices also influence overall bacterial numbers. Our findings illustrate that specific dimension of variability, namely total copy number, which could be applied as a proxy of total bacterial biomass or absolute bacterial abundance. We speculate that the total bacterial biomass may be important in microbiome function and that acute contractions in overall magnitude (e.g. due to antibiotics) could be important for interspecies competition and changes in dominance within the community. Furthermore, across a group of infants at any given timepoint, typical indices of biodiversity (e.g. alpha and beta diversity) may be somewhat misleading due to the large variability in overall microbiome load between infants. In essence, one could have high diversity, but as part of an overall very low microbial load. Thus, it highlights a pitfall in the amalgamation of relative abundance data from groups of individuals, even those with similar clinical courses and is particularly applicable to this very vulnerable group where the microbiome is just being established.

An inherent limitation of this approach is inter-species variability in gene copy number (GCN). Genera with high GCNs (e.g. *Escherichia* (*E. coli* GCN = 7)) may be overestimated compared to low-GCN genera (e.g. *Bifidobacterium (B. bifidum* GCN = 2)). This GCN bias primarily affects between-taxa comparisons but has minimal impact on within-taxon comparisons across samples, and bias is reduced by analysis at genus rather than species level. We acknowledge the limitations of and inaccuracy introduced by this technique but argue that the results of this study remain valid. Indeed, important findings such as the substantial effect of antibiotic treatment on *Bifidobacterium* may be underestimated.

Importantly, this work has demonstrated the pronounced effect of antibiotic administration on genera within the neonatal gut microbiome, most notably *Bifidobacterium*. Given the prevalence of use of *Bifidobacterium* sp. in current neonatal probiotics and their perceived importance in prevention of adverse neonatal outcomes,^21^ development of strategies to increase their fitness within the microbiome are essential.

In the future, utilization of methods to estimate overall microbiome magnitude may reveal important considerations in evaluation of the gut microbiome at this early stage of establishment, particularly in preterm infants. We also suggest that future studies could incorporate GCN correction factors or metagenomic sequencing to further improve quantification. These techniques may help to improve our understanding of longitudinal community dynamics within the neonatal gut microbiome and add to our knowledge of the role of the microbiome in complications of prematurity.

## Declaration

The data that support the findings of this study are openly available in the National Centre for Biotechnology Information Sequence Read Archive. BioProject ID PRJNA1216257.
